# Complete Remission in Locally Advanced Breast Cancer: What Comprehensive Multi-Modality Treatment Has to Offer in Sub-Saharan Africa

**DOI:** 10.7759/cureus.1432

**Published:** 2017-07-06

**Authors:** Gaurav Bhattacharya, Susan C Msadabwe-Chikuni, Roanne Segal, Omkar Inamdar, Catherine K Mwaba

**Affiliations:** 1 Radiation Oncology, The Ottawa Hospital Cancer Centre, The University of Ottawa; 2 Clinical and Radiation Oncology, Cancer Diseases Hospital, Lusaka, Zambia; 3 Medical Oncology, The Ottawa Hospital Cancer Centre, The University of Ottawa

**Keywords:** locally advanced, breast cancer, low resource, clinical outcome

## Abstract

Locally advanced breast cancer presents as a heterogeneous disease, but it is often best treated with aggressive combined modality therapy.  Commonly, it carries a more guarded prognosis. Given the above, it can be a particularly challenging entity to treat in resource-limited settings.

We identify one such case with a relative lack of hormone receptor positivity in the sub-Saharan country of Zambia. Management of the disease was hampered by the challenges of resource constraints and communication gaps that are especially acute in low- to middle-income nations as compared to Western societies. However, with skilled interdisciplinary advice and the means available at a tertiary care facility, our patient was able to afford a superior clinical outcome in the form of a pathologic complete response via the use of surgical, systemic, and radiotherapy modalities. Additionally, the ensuing remission was corroborated by a careful follow-up regime.

We thus reinforce the feasibility and value of a team-based approach in the management of this disease regardless of the setting.

## Introduction

Major advances have been seen in the detection, treatment, and cure of patients with breast cancer (BC) over the last decade. Despite this, BC still affects over two million women worldwide [1]. To date, the etiology of BC remains unclear, but several epidemiological factors including advanced age, family history, early menarche, nulliparity, and late menopause have been considered as risk factors. In North America and Europe, where screening programs exist, the majority of women with early-stage disease are detected, and it is treated with curative intent with overall survival estimates being as high as 80%-90% [1]. On the other hand, in a resource-limited sub-Saharan country such as Zambia, most patients present with locally advanced or metastatic disease although data around incidence, prevalence, and the stage of breast cancer can be challenging to quantify. For example, in Zambia, only 10%-15% of cancer diagnosis are captured nationwide [2], suggesting an underestimation of actual figures [3-4]. Nevertheless, BC represents approximately 11% of all cancers recorded, constituting a major national concern [2-5] with the majority presenting with either Stage III or IV disease [6].

In this scenario, we highlight locally advanced breast cancer (LABC), defined as tumors greater than 5 cm with regional lymphadenopathy, or those with direct chest wall extension with or without skin involvement. While still considered to be potentially curable, generally speaking, they are more difficult to treat and require combined modality therapy and have had historically poorer outcomes. Here, we provide evidence of a clinical case report with a favorable outcome, demonstrating the feasibility of treating LABC in an integrated multidisciplinary collaboration in a resource-limited setting.  

## Case presentation

A 30-year-old Zambian female (AB) first identified a small lump in her right breast on breast self-examination in August 2012.  It was associated with generalized local swelling and features consistent with mastitis. At the time, no nipple discharge, or any other concerning features including constitutional symptoms, were present. There was no family history of malignancy. She was premenopausal, nulligravida, and worked as a nurse in the Mazabuka District Hospital. A single course of oral antibiotics was prescribed without effect. Subsequently, the mass progressed over a four-month period. An ultrasound guided “biopsy” (from here on referred to as lumpectomy) was undertaken on 14 November 2012. The pathology showed two masses identified within the specimen, the first representing the breast primary, with the second lesion from the axilla. The breast specimen measured 5 x 4 x 2 cm and the axillary mass 4 x 2 x 1 cm. Both histological specimens were consistent with infiltrating breast carcinoma of ductal origin. There was no mention of grade or surgical margins. Tumor markers were estrogen receptor (ER) negative and progesterone receptor (PR) positive with high intensity in 30% of the lesion. Unfortunately, owing to reagent shortage in the private laboratory, HER-2 staining was not performed.

On 22 November 2012, she was referred to the tertiary-level teaching hospital in Lusaka (University Teaching Hospital) as well as the Cancer Diseases Hospital, which is the only oncology center in the country, for consideration of radical therapy. At presentation, her clinical examination showed a right breast infra areolar lumpectomy scar measuring 3 x 4 cm with associated skin desquamative changes (no peau d’orange; the skin involvement having been removed during the prior lumpectomy). In addition, a 1 cm mobile axillary lymph node was identified. She was consequently staged as clinical T4B N1 (Stage IIIB) disease. Subsequent imaging in December included a chest X-ray and an abdominal ultrasound which were both normal. Laboratory testing included full blood count with differential, and electrolytes and liver function tests; all of which were within normal limits.

Given her young age, large primary, positive nodal status, and relative lack of hormone receptor positivity, she was offered neoadjuvant TAC (docetaxel at 75 mg/m², doxorubicin at 50 mg/m², and cyclophosphamide at 500 mg/m²) chemotherapy every 21 days for a planned six cycles. This was completed on 27 March 2013.  She did not have any significant toxicity. Remarkably, by Cycle 6, she had a complete clinical response within the breast as well as the nodes. No additional imaging was later performed.

On 23 April 2013, she was taken to the operating theatre for a complete right simple mastectomy. This included skin with nipple and breast fat extending 6 cm as well as a Level III axillary nodal dissection. The intraoperative report highlighted that “No mass was noted. Within the axilla, a few suspicious masses query lymph nodes.” These nodes were targeted for pathological review.  Subsequently, she had an unremarkable post-op recovery.

**Figure 1:**
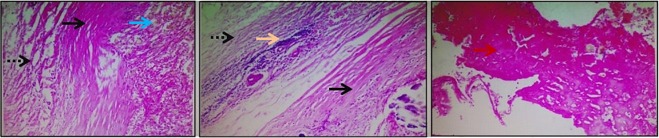
Mastectomy specimen Hematoxylin-eosin (H&E) stained sections of the mastectomy specimen show areas of hemorrhage (red arrows), fibrosis (black arrows), and focal inflammatory infiltrate (white arrows) with focal necrosis, consistent with tumor bed and the previous surgical procedure. Hemosiderin-laden macrophages (blue arrows), dilated blood vessels, and chronic inflammatory cells are present in the stroma. No evidence of viable tumor cells is seen.

The final pathological response highlighting the occasional focus on fibrosis and mild chronic inflammation as well as a normal appearing nipple with pagetoid spread of disease (Figure 1) did not demonstrate any residual disease within the breast or the axillary nodes and was consistent with a complete pathological response. 

Faced with a relatively challenging treatment timeline, the case was discussed at local tumor board, and a decision was made to proceed with adjuvant radiation based on established guidelines [7]. External beam radiotherapy comprising of 50 Gy in 25 fractions using 6 MV photons via the local linear accelerator to a 3 cm depth was prescribed using three tangential beams with two-dimensional planning. This was followed with a boost of 10 Gy in 2 Gy fractions via 7 MeV electrons for boost treatment to the surgical scar site. Once again, infrastructure challenges including frequent machine breakdowns and extensive wait times meant that the first fraction was not delivered before July 2013 and was completed only by 8 August for a total of nine months from lumpectomy to completion of radical treatment.

Given the positive post-radiation status, she also started on tamoxifen on 7 May 2013, for a planned duration of at least five years. As of January 2017, she remained on a surveillance program and no evidence of disease recurrence or metastases has been identified. A timeline summary table with extensive details is provided below.

**Table 1:** Case summary

Dates (dd/mm/yyyy)	Symptoms/Findings	Diagnosis	Treatment	Outcome
08/2012	Right breast lump	Mastitis	Antibiotics	Progression of mass
14/11/2012	5 x 4 x 2 cm breast specimen, 4 x 2 x 1 cm axillary mass	Invasive ductal carcinoma		Referral to tertiary-level hospital
22/11/2012	Right breast infra areolar lumpectomy scar (3 x 4 cm) with associated skin desquamative changes, 1 cm mobile axillary lymph node	Ibid: cT4B cN1 disease		Proceed to completion of remaining staging exams
06/12/2012	Negative abdominal ultrasound			
08/12/2012	Negative chest X-ray	M0 (negative for systemic metastases): Stage IIIB		Proceed to radical treatment
11/12/2012- 27/03/2013			Six cycles of neoadjuvant TAC chemotherapy	Clinical complete response
23/04/2013			Completion right simple mastectomy plus level III axillary nodal dissection	Pathologic complete response
22/01/2013		Chemotherapy-induced menopause identified		
07/05/2013			Tamoxifen initiated (five years)	
25/05/2013				Tumour board discussions and proceed to adjuvant radiotherapy
08/07/2013- 09/08/2013			50 Gy/25 fractions plus 10 Gy/5 fractions boost to scar	Wet desquamation of skin plus chest wall pain (resolved post topical steroids, postoperative analgesic treatments respectively)
03/12/2013	Negative mammogram			
04/09/2013- 25/1/2017	No clinical evidence of recurrence or metastases			Continue followups every 6-12 months

## Discussion

The challenges faced in the case of AB are manifold. A lack of electronic medical record systems, unavailability of modern facilities (such as 3-dimensional radiotherapy planning), shortage of trained personnel (only nine out of 52 African countries have training programs for radiation oncologists and radiation and medical physicists; Zambia is not one amongst these nine countries), resource constraints (shortage of reagent availability and pharmacy chemotherapy), and systemic inefficiencies (delayed time span between pathological diagnosis and treatment end time) are some of the challenges facing the delivery of oncologic care in healthcare systems across sub-Saharan Africa.

Another point of note is that Zambian mammography guidelines have historically been guided by the American Cancer Association guidelines for screening. At present, a baseline study is initiated between ages 35 and 39 followed by annual or biennial imaging between the ages of 40 and 49 and finally annually at ages 50 and upwards. Therefore, the diagnosis of patient AB could not have been picked up, regardless of screening measures. As an aside, more research needs to probably be conducted in regards to the age of baseline screening in Zambia.

That being said, several important learning points may be garnered from this case. Long-term remission of LABC requires access to tertiary-level care. The ability of the healthcare system to transition referral services (both private laboratories and distant government hospitals contributed to her eventual remission), provision of experienced surgical facilities, and access to expert oncologic tumor boards at the Cancer Diseases Hospital (the only oncologic specialty center for all of Zambia as well as Zimbabwe and Malawi), and government investment in essential infrastructure are to be lauded. The availability of radiotherapy in Zambia remains a boon in this case. Indeed, 29 African nations do not possess any radiotherapy facilities at all. These resource constraints need to be overcome with further judicious government healthcare spending (Zambia’s healthcare budget in 2016 actually declined from 9.6% to 8.3% of the total national budget from the previous year) [8]. Increased awareness of screening programs and international initiatives such as the Global Impact of Radiation in Oncology (GIRO) partnership from the European Society for Radiotherapy & Oncology (ESTRO) (which aims to save a million lives by 2035), and even private corporation derived initiatives such as the Virtual University for Cancer Control Africa project led by the Roche African Research Foundation, the United States Government, and the International Atomic Energy Agency can also assist in such an endeavor [9].

Finally, patient-clinician rapport is also critically important to ensure not just adherence to the prescribed therapeutic regimen but also to ensure post-treatment surveillance. A lack of a multi-tier care provider model in Zambia means that cancer patients routinely see their oncologists for even relatively banal non-oncologic care. For example, in the case of AB, gout was diagnosed incidentally during radiotherapy and a nonsteroidal anti-inflammatory drug (NSAID) was prescribed by her clinical oncologist. This level of medical commitment cements the therapeutic relationship despite the wait time challenges considered routine in the sub-Saharan medical system.

## Conclusions

The continuing remission of LABC in this young female patient with negative estrogen receptor status highlights that despite the inherent obstacles in a low-income country, judicious use of even relatively antiquated resources in a guidelines-based practice can make a significant impact at the individual level. We advocate multi-disciplinary collaboration between experienced oncologic teams and dedicated tumor board discussions in order to continue with such excellent clinical outcomes. 
